# ProstateZones – Segmentations of the prostatic zones and urethra for the PROSTATEx dataset

**DOI:** 10.1038/s41597-024-03945-2

**Published:** 2024-10-08

**Authors:** William Holmlund, Attila Simkó, Karin Söderkvist, Péter Palásti, Szilvia Tótin, Kamilla Kalmár, Zsófia Domoki, Zsuzsanna Fejes, Zsigmond Tamás Kincses, Patrik Brynolfsson, Tufve Nyholm

**Affiliations:** 1 Umeå University, Department of Diagnostics and Intervention, Umeå, Sweden; 2 University of Szeged, Albert Szent-Györgyi Medical School, Department of Radiology, Szeged, Hungary; 3 Skåne University Hospital, Department of Haematology, Oncology and Radiation Physics, Lund, Sweden

**Keywords:** Prostate, Magnetic resonance imaging

## Abstract

Manual segmentations are considered the gold standard for ground truth in machine learning applications. Such tasks are tedious and time-consuming, albeit necessary to train reliable models. In this work, we present a dataset with expert segmentations of the prostatic zones and urethra for 200 randomly selected patients from the PROSTATEx dataset. Notably, independent duplicate segmentations were performed for 40 patients, providing inter-reader variability data. This results in a total of 240 segmentations. This dataset can be used to train machine learning models or serve as an external test set for evaluating models trained on private data, thereby addressing a current gap in the field. The delineated structures and terminology adhere to the latest Prostate Imaging Reporting and Data Systems v2.1 guidelines, ensuring consistency.

## Background & Summary

Prostate cancer is the most prevalent malignancy in males worldwide^1^, requiring accurate diagnostic and treatment approaches. For diagnosis, the global Prostate Imaging Reporting and Data Systems (PI-RADS) guidelines are used to assess prostatic lesions on multi-parametric magnetic resonance imaging (mpMRI), with different primary sequences depending on their zonal location^2^. These anatomical zones of the prostate are defined as the peripheral zone (PZ), central zone (CZ), transitional zone (TZ), and anterior fibromuscular stroma (AFS), and presents different characteristics and histological features^3^. Radiotherapy treatment of prostate cancer has traditionally been delivered with a homogeneous dose to the entire prostate. Recent studies have demonstrated the efficacy of a local dose escalation to a sub volume of the prostate, a so-called focal boost^4^. The relationship between urethral dose and urinary toxicity highlights the importance of limiting the dose to the intraprostatic urethra to minimize side effects from treatment^5,6^. Additionally, focal boost treatment could potentially be further optimized by using the zonal information to individualize treatment and risk stratification, as the location of the cancer in different zones has different incidence, prognosis, and outcome, making treatment zonal-dependent instead of zonal-agnostic^7^.

Manual segmentations of the prostate, its anatomical zones, and the urethra on MRI are tedious and time-consuming. Therefore, the development of an individualized, automatic method to segment the prostate, its internal zones, and the urethra is relevant in current medical practice, with implications both for treatment and diagnosis. The current literature of machine learning methods for automatic segmentations of prostate zonal anatomy on MRI was recently discussed in a topical review^8^. The review highlighted current limitations in clinical applicability regarding the data used. Terminology and delineated structures were inconsistent, inter-reader variability to compare model performance was generally lacking, and most critically, an open external dataset for comparison of model performances was absent.

In this work, we present a dataset with manual segmentations of the prostate and its internal zones, with terminology consistent with the PI-RADS v2.1s guidelines, as well as the prostatic urethra. It comprises 240 segmentations for 200 patients from the PROSTATEx dataset^9^, totalling 1200 individual structures. For 40 patients, independent duplicate segmentations are provided, presenting inter-reader variability data useful for comparing the performance of automatic segmentation models. This dataset can be used either as a training and testing dataset for machine learning purposes or as an external dataset for models trained on private datasets to allow for unbiased testing of model performances.

To the best of our knowledge, it is the first publicly available dataset containing all the prostatic zones and the first publicly available dataset containing the prostatic urethra delineated on MRI.

## Methods

### Image data

The dataset consists of 200 patients, programmatically randomly selected, from the PROSTATEx dataset available at the Cancer Imaging Archive^9,10^. Each sample contains mpMRI exams acquired from one of two different 3 T MR Siemens scanners (MAGNETOM Trio and Skyra). T2-weighted (T2w) images were acquired with an in-plane resolution of 0.3 to 0.7 mm and a slice thickness of 3.0 to 4.0 mm, with 0.5 × 0.5 × 3.0 mm being the most frequent resolution (74%). The selected data consisted of patients with (66) and without (134) clinically significant prostate cancer (csPCa), defined as a biopsy proven Gleason Grade Group ≥2. Other image sequences are available, as well as patient selection criteria^11^.

### Segmentations

The prostatic zones and urethra were manually segmented slice by slice on the axial T2-weighted images by two experienced radiologists working in collaboration with three junior colleagues. The two more experienced radiologists had >1000 and ~500 interpreted prostate MRIs each, while the juniors had <100 prostate MRIs each. All delineations by junior colleagues were checked and, if necessary, corrected by one of the more experienced radiologists. The delineations were based on the zonal description by McNeal^3^ and the PI-RADS guidelines (v2.1)^2^. A retrospective dose analysis for focal boost treatment was used as the basis for the urethra^12^. A short description of the segmented structures is provided in Table 1.

**Table 1:** Structure descriptions.

Region of interest	Description
Peripheral Zone (PZ)	Covers the outer lateral and posterior regions; makes up most of apex of the prostate. Appears homogeneously hyperintense on T2w MRI.
Central Zone (CZ)	Originates from the verumontanum and surrounds the ejaculatory ducts proximally; extends as an inverted cone in the coronal view from its origin towards the base of the prostate. Appears homogeneously hypointense on T2w MRI.
Transitional Zone (TZ)	Consists of two independent lobes along the proximal prostatic urethra; enlarges with ageing due to benign prostatic hyperplasia (BPH), which can affect the appearance of all zones of the prostate. Demonstrates heterogeneous signal intensity on T2w MRI.
Anterior Fibromuscular Stroma (AFS)	Contains smooth muscle and no glandular tissue; extends almost as an apron from the bladder neck over the anteromedial surface of the prostate. Appears hypointense on T2w MRI.
Urethra	The part of the urethra included within the prostate contour. Delineated as a circular structure with a 6 mm diameter in the axial plane.

The delineation procedure was performed with 3D Slicer^13^ v.5.2.2 and started by initially delineating the prostate contour followed by the prostatic urethra, as a circular structure with a 6 mm diameter in each slice. Thereafter, the prostatic zones were generally delineated in the order of PZ, TZ, CZ, and AFS. If the urethra was difficult to delineate in any individual slice, it was considered acceptable to interpolate that slice based on adjacent slices if the result was corrected to follow the desired structure.

Out of the total 200 patients selected, 40 were delineated independently by two experienced radiologists. This resulted in two independent segmentations for all test patients, creating an inter-reader variability baseline, which allows for a comparison of the model performance. After completion, all segmentations were reviewed by a multi-professional team, who performed minor adjustments, removing isolated pixels and harmonizing segmentations between slices in Hero v.2023.1.1 (Hero Imaging AB, Umeå, Sweden; https://www.heroimaging.com/). Two different individuals performed the minor adjustments for the duplicate segmentations to ensure reliability. Subsequently, all segmentations were submitted for final approval from one of the two experienced radiologists, with the duplicates approved by the initial delineator using the online evaluation tool naesView (https://naesview.com). In the end, the resulting dataset includes 160 samples with single segmentations for training, and 40 samples with duplicate segmentations for testing.

## Data Records

The dataset is available at Zenodo^14^.

Each patient segmentation is saved as an nrrd-file which contains image geometry but no patient metadata. The segmentations have names corresponding to each respective patient following the PROSTATEx naming convention. In short, the segmentation for patient 0000 is called *Seg-0000*, and duplicate segmentations are separated between reader 1 and 2 as *Seg-####_R1* and *Seg-####_R2*, respectively. If metadata is needed, it can be gathered from the corresponding PROSTATEx DICOM images.

A summary file containing information from PROSTATEx about the presence of csPCa in the samples included in this cohort is available in Excel format. Full information is available at the image repository^9^.

## Technical Validation

**Fig. 1:**
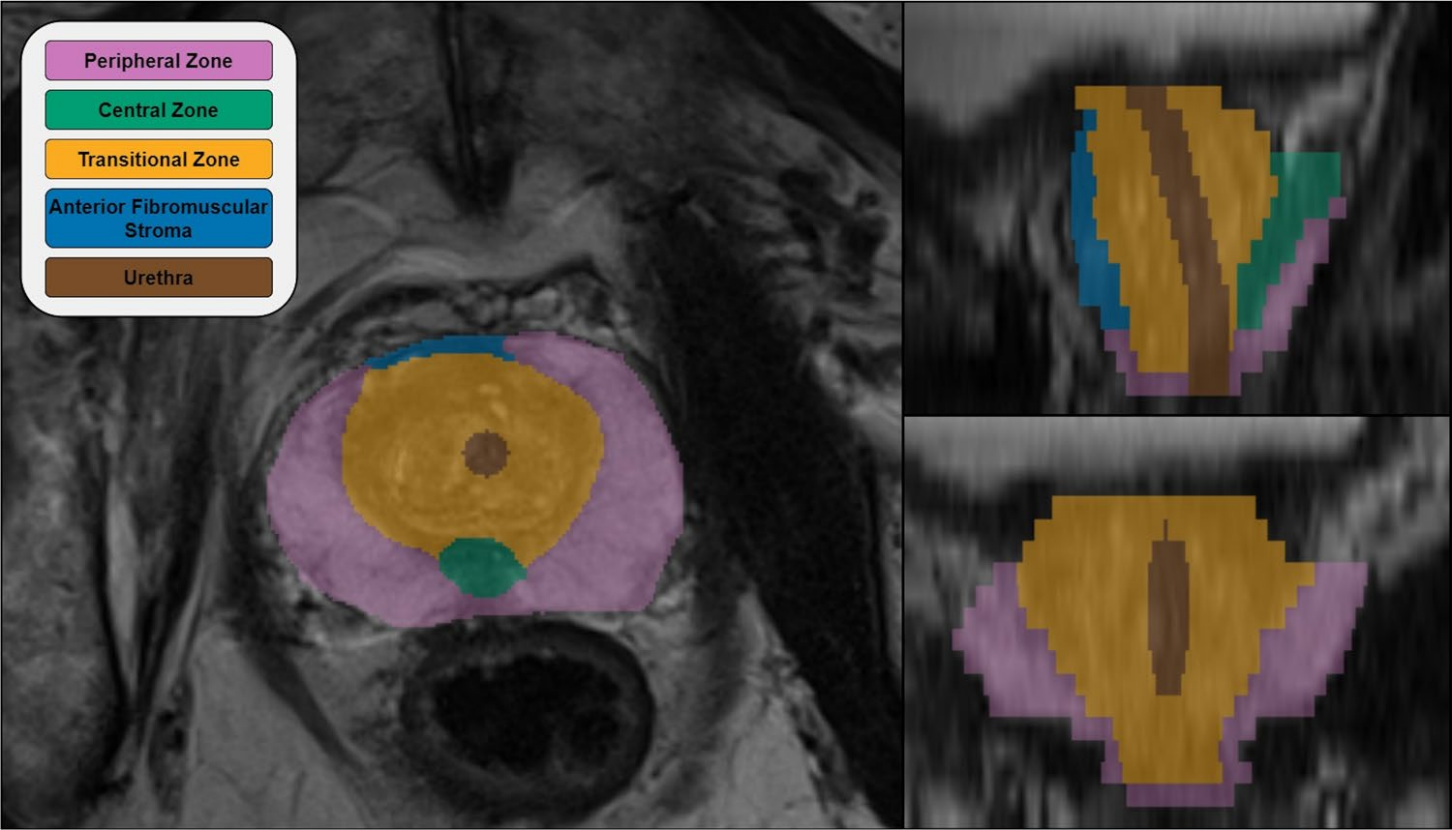
Example segmentation. The structures delineated for one patient displayed in the axial (left), sagittal (top right) and coronal view (bottom right).

An example of the delineated structures for a representative patient is displayed in Fig. 1.

For the 40 duplicate samples, inter-reader variability is presented as *mean ± standard deviation* in Table 2. The inter-reader variability metrics show overlap and boundary measures, with Dice Similarity Coefficient (*DSC*) as well as Hausdorff Distance (*HD*) and Average Symmetric Surface Distance (*ASSD*)^15^. Additionally, the Centre Line Distance (*CLD*) is provided for the urethra. All metrics are volumetrically based (i.e. not measured slice-wise) and provide context for the consistency of the delineations and can be used when comparing the performance of automatic segmentation methods.

- Dice Similarity Coefficient (*DSC*):
  The *DSC* measures the volumetric overlap between a reference mask and a prediction,
  $${DSC}=\frac{2\left|{V}_{{\rm{ref}}}\cap {V}_{{\rm{pred}}}\right|}{\left|{V}_{{\rm{ref}}}\right|+\left|{V}_{{\rm{pred}}}\right|}$$
  where ${V}_{{\rm{ref}}}$ and ${V}_{{\rm{pred}}}$ represent the reference and predicted volumes, respectively.
  Hence, *DSC* is one for a complete overlap between the two volumes and zero for no overlap.
- Hausdorff Distance (*HD*):
  The *HD* is a boundary metric that calculates the maximum of all shortest distances for all voxels from one surface to the other,
  $${HD}({S}_{{\rm{ref}}},{S}_{{\rm{pred}}})=\max (h\left({S}_{{\rm{ref}}},{S}_{{\rm{pred}}}\right),h\left({S}_{{\rm{pred}}},{S}_{{\rm{ref}}}\right))$$
  where ${S}_{{\rm{ref}}}$ and ${S}_{{\rm{pred}}}$ are the reference and predicted surfaces, respectively, as derived from the original volumes, and $h({S}_{{\rm{ref}}},\,{S}_{{\rm{pred}}})$ is given by,
  $$h({S}_{{\rm{ref}}},{S}_{{\rm{pred}}})=\mathop{\max }\limits_{r{\rm{\in }}{S}_{{\rm{ref}}}}\,\mathop{\min }\limits_{p{\rm{\in }}{S}_{{\rm{pred}}}}||r-p||$$
  where $|\left|r-p\right||$ is the Euclidean distance.
  Ideally, *HD* should be as close to zero as possible, but it is sensitive to outliers as the maximum distance of all shortest distances between the surfaces is returned.
- Average Symmetric Surface Distance (*ASSD*):
  The *ASSD* measures the average of all distances for every voxel from one surface to the other and vice versa. It is given by,
  $${ASSD}\left({S}_{{\rm{ref}}},{S}_{{\rm{pred}}}\right)=\frac{d\left({S}_{{\rm{ref}}},{S}_{{\rm{pred}}}\right)+d({S}_{{\rm{pred}}},{S}_{{\rm{ref}}})}{{N}_{{\rm{ref}}}+{N}_{{\rm{pred}}}}$$
  where ${S}_{{\rm{ref}}}$ and ${S}_{{\rm{pred}}}$ as well as ${N}_{{\rm{ref}}}\,$ and ${N}_{{\rm{pred}}}$ are the reference and predicted surfaces and their number of surface voxels, respectively. The distance $d({S}_{{\rm{ref}}},{S}_{{\rm{pred}}})$ is determined as,
  $$d({S}_{{\rm{ref}}},{S}_{{\rm{pred}}})=\sum _{r\in {S}_{{\rm{ref}}}}\,\mathop{\min }\limits_{p{\rm{\in }}{S}_{{\rm{pred}}}}||r-p||$$
  where $|\left|r-p\right||$ is the Euclidean distance.
  Like *HD*, *ASSD* would ideally be as close to zero as possible, although it is less sensitive to outliers as it represents an average over all shortest distances between the voxels of the two surfaces.
- Centre Line Distance (*CLD*):

**Table 2:** Inter-reader variability metrics for the 40 duplicate samples. Presented as mean ± standard deviation.

Zone	*DSC*	*HD* [mm]	*ASSD* [mm]	*CLD* [mm]
Prostate	0.913 ± 0.027	5.9 ± 1.4	0.87 ± 0.21	—
PZ	0.751 ± 0.054	10.8 ± 3.9	1.08 ± 0.25	—
CZ	0.43 ± 0.15	14.2 ± 4.7	3.3 ± 1.4	—
TZ	0.826 ± 0.062	7.7 ± 2.2	1.25 ± 0.31	—
AFS	0.37 ± 0.12	10.3 ± 4.6	1.97 ± 0.72	—
Urethra	0.35 ± 0.14	7.8 ± 2.8	2.15 ± 0.79	3.6 ± 1.1

The *CLD* is calculated as the *ASSD* for two skeletonized structures, meaning the delineations are reduced to a single voxel per slice.

The *CLD* should ideally be as close to zero as possible as this indicates smaller deviations between the skeletonized delineations.

For a more detailed analysis of the inter-reader variability, a confusion matrix is presented in Table 3. The matrix shows the segmentations of Reader 1 along the rows and Reader 2 along the columns. Discrepancies are indicative of individual tendencies in the segmentation approach of each reader. Reader 1 tends to delineate a prostate that is 5% larger than that delineated by Reader 2. This enlargement is predominantly observed in the size of the PZ and CZ, which explains most of the differences observed in the matrix for these structures and the background. Reader 2 outlines a larger AFS, which then overlaps with TZ as classified by Reader 1. Other discrepancies have less individual tendencies, and most disagreements classified as adjacent structures, presumably along the borders. This is an anticipated outcome given the absence of visually distinct boundaries, underscoring the complexity of the task.

**Table 3:** Confusion matrix derived from the inter-reader variability of the duplicate samples (n = 40), with the segmentations of Reader 1 represented along the rows and Reader 2 along the columns.

	Background	PZ	CZ	TZ	AFS	Urethra
Background	2118093	1541	39	1382	370	10
PZ	2660	13319	377	2288	29	34
CZ	1019	352	1284	473	0	6
TZ	2047	1106	569	30317	866	706
AFS	352	11	0	282	594	0
Urethra	60	26	247	447	1	400

The data is presented as a per patient average in units of mm^3^.

## Usage Notes

For the simplest usage of the dataset, a description with Python code is available (https://github.com/UMU-DDI/ProstateZones) of how to extract the relevant images from the PROSTATEx dataset and to set up a folder structure containing images and their corresponding segmentations upon download.

## Data Availability

No code is needed to use the data, but a Python script for simple structuring is available (https://github.com/UMU-DDI/ProstateZones). The GitHub repository includes a requirements .txt file, a Python script, as well as other complementary files. Additionally, the GitHub repository includes the Hero workflow used to calculate the inter-reader variability metrics for full transparency.
